# Characterizing Middle Eastern and North African Patients in US Transplant Data

**DOI:** 10.1097/TXD.0000000000001973

**Published:** 2026-07-07

**Authors:** Jesse Howell, Jesse D. Schold, Lisa M. McElroy, Oscar K. Serrano, Alejandro Diez, Erin Schnellinger

**Affiliations:** 1 Research Department, United Network for Organ Sharing, Richmond, VA.; 2 Department of Surgery-Transplant, University of Colorado Anschutz Medical Campus, Aurora, CO.; 3 Departments of Surgery and Population Health Sciences, Duke University, Durham, NC.; 4 Hartford Hospital Transplant Program, Hartford, CT.; 5 University of Connecticut School of Medicine, Farmington, CT.; 6 Department of Internal Medicine, The Ohio State University College of Medicine, Columbus, OH.

## Abstract

**Background.:**

The Office of Management and Budget’s March 2024 Statistical Policy Directive 15 revision requires reporting Middle Eastern or North African (MENA) individuals separately from the White category. This study compares MENA and non-MENA transplant candidates/recipients before the revision to determine whether differences exist between these populations.

**Methods.:**

We analyzed Organ Procurement and Transplantation Network (OPTN) single-organ kidney, liver, heart, and lung registrations ever-waiting or transplanted between January 1, 2018, and June 30, 2023, filtering to White, non-Hispanic (NHW) US residents. Individuals reporting Arab/Middle Eastern and North African race/ethnicity were classified as MENA. Demographics, 1-y Kaplan–Meier graft/patient survival, transplant and waitlist mortality rates, and median time to transplant (based on cumulative incidence) were calculated.

**Results.:**

MENA registrations were younger (7.0% versus 3.3%; <18 y), more frequently male (71.4% versus 63.8%), and more commonly kidney candidates (70.1% versus 59.7%), on public/charity insurance (60.6% versus 48.8%). MENA kidney registrations had longer median dialysis time at listing (154 versus 47 d) compared with non-MENA registrations. Larger proportions of MENA liver registrations were listed under the least medically urgent statuses, while larger proportions of MENA thoracic registrations were listed under more medically urgent statuses. Median time to transplant was longer for MENA versus non-MENA liver registrations (662 versus 443 d), and shorter for MENA versus non-MENA thoracic registrations (64 versus 83 d). One-year graft/patient survival was similar (eg, liver recipients: 0.95 versus 0.94). Transplant and waitlist mortality rates were lower for MENA versus non-MENA liver candidates (69.4 versus 89.2 deceased donor transplants (7.0 versus 11.0 waitlist deaths) per 100 active or total patient-years, respectively).

**Conclusions.:**

Dialysis time at listing, medical urgency distributions, waitlist mortality rates, transplant rates, and median time to transplant differed between MENA and non-MENA candidates. As more data accrue, separate monitoring of MENA candidates/recipients may further elucidate the waitlist and transplantation experience of these individuals.

The March 2024 revision of the Office of Management and Budget’s Statistical Policy Directive No. 15 (SPD 15^1^) created a new reporting category for Middle Eastern or North African (MENA) individuals, distinct from the “White” category. The purpose of this change was to reflect the tendency of many MENA individuals to not identify as White and to identify potential variations in health care access or outcomes that MENA individuals might experience.^2,3^ These changes affect federal data collection, including data collected by the Organ Procurement and Transplantation Network (OPTN) to facilitate organ transplantation. In anticipation of these changes, this study compares demographic and clinical characteristics of MENA and non-MENA individuals who were waiting for or received a solid organ transplant before the SPD 15 revision to determine whether differences exist between these populations.

## MATERIALS AND METHODS

This study used data from the OPTN. The OPTN data system includes data on all donors, wait-listed candidates, and transplant recipients in the United States, submitted by the members of the OPTN. The Health Resources and Services Administration (HRSA), US Department of Health and Human Services, provides oversight to the activities of the OPTN contractor. Institutional Review Board (IRB) exemption was obtained from HRSA.

We analyzed single-organ kidney, liver, heart, and lung registrations ever-waiting on the OPTN waitlist or transplanted between January 1, 2018, and June 30, 2023. Heart and lung were combined into 1 thoracic category given small numbers. We filtered to the White, non-Hispanic (NHW) category, defined as those who indicated White race (and no other racial group) on the Transplant Candidate Registration Form (TCR) or Transplant Recipient Registration Form (TRR) along with either “not Hispanic or Latino” or “Ethnicity not reported” for candidate ethnicity. Analyses were restricted to US residents, which includes US citizens and noncitizens residing in the United States, as about 20% of MENA candidates on the OPTN waitlist were non-US residents with inherently different demographics (**Table S1, SDC**, https://links.lww.com/TXD/A879). NHW candidates and recipients who reported Arab/Middle Eastern and North African race/ethnicity on the TCR or TRR were classified as MENA. All other NHW candidates and recipients were classified as non-MENA (**Figure S1, SDC**, https://links.lww.com/TXD/A879). Note that the transplant cohort included all transplants that occurred between January 1, 2018, and June 30, 2023, and was not simply the subset of ever-waiting candidates who received a transplant. Creating separate waiting list and transplant cohorts ensured that all MENA and non-MENA candidates and recipients would be captured, including those who may have indicated a different race/ethnicity on their TCR versus TRR forms. For more details on the construction of the ever-waiting and transplant cohorts, please see the (Supplemental Digital Content, SDC, https://links.lww.com/TXD/A879).

All analyses for candidates were performed at the registration level, with the exception of mortality rates, which were calculated at the patient level. The following demographic characteristics were examined across MENA groups for ever-waiting and transplant cohorts: organ (kidney, liver, thoracic), age (years), birth sex, OPTN region, insurance (private or self-pay versus public or charity); the public or charity category includes Medicaid, Medicare FFS (Fee for Service), Medicare & Choice, CHIP (Children’s Health Insurance Program), Department of Veterans Affairs, Other government, Donation, Free Care, Foreign Government Specify, Public insurance—Medicare Unspecified, US/State Government Agency), body mass index (BMI, kg/m^2^), and prior transplants. Donor type was examined for the transplanted cohort only. The deceased donor category included both US deceased donors and foreign deceased donors. One-year Kaplan–Meier graft and Kaplan–Meier patient survival were estimated. Medical urgency was calculated using waiting list area under the curve (WLAUC) for lung, model for end-stage liver disease (MELD)/pediatric end-stage liver disease model (PELD) for liver, heart statuses for heart, and dialysis time at listing for kidney. Registrations were excluded from the medical urgency table if they had a heart or liver status at listing or transplant from a previous allocation system (ie, a heart status from before the 2018 heart allocation change^4^ or a liver status preceding the initial implementation of MELD/PELD in 2002^5^). Dialysis time at listing was calculated for kidney registrations as the difference between OPTN waitlist registration date and dialysis start date, with preemptively listed candidates set to 0. Sensitivity analyses removed anyone who was not on dialysis at listing. Rates were calculated as the number of deceased donor transplants per 100 active patient-years (since inactive registrations are ineligible to receive a transplant) or the number of waiting list deaths (including deaths within 14 d of removal) per 100 total patient-years. For patient-level analyses, candidates were considered MENA if any of their registrations indicated Arab/Middle Eastern or North African race/ethnicity. Median time to transplant was calculated for liver and thoracic registrations by estimating the cumulative incidence of deceased donor transplant (while accounting for the presence of other competing waiting list outcomes) and identifying the first time point at which the probability of deceased donor transplant reaches or exceeds 50%. Median time to transplant was unable to be calculated for kidney registrations as less than 50% were transplanted in the cohort period.

Analyses were performed using R 4.3.3^6^ and SAS Enterprise Guide 8.2.^7^ Analyses are based on OPTN data as of August 21, 2025, and are subject to change based on future data submission or correction.

## RESULTS

There were 207 136 NHW registrations (183 954 distinct patients) in the waitlist cohort; 3114 (1.5%) registrations were MENA, representing 2869 distinct MENA patients. MENA registrations tended to be younger, with a larger proportion of MENA registrations listed below 18 y of age (7.0% versus 3.3%) and a smaller proportion of MENA registrations listed at or above 65 y (22.3% versus 26.0%). Additionally, higher proportions of MENA registrations were kidney registrations (70.1% versus 59.7%), male (71.4% versus 63.8%), had lower BMI (32.7% versus 28% <25 kg/m^2^ BMI), and were on public or charity insurance (60.6% versus 48.8%) compared with non-MENA registrations (Table 1). Median dialysis time at listing for MENA kidney registrations was 154 d (interquartile range [IQR]: 0–568), compared with 47 d (IQR: 0–437) for non-MENA kidney registrations. Sensitivity analyses yielded the same directionality but a smaller magnitude of difference in median dialysis time between groups (MENA: 489 d [IQR: 232–935] versus non-MENA: 418 d [IQR: 213–833]). A higher proportion of MENA liver registrations were listed under the least medically urgent category (ie, MELD/PELD < 15), whereas higher proportions of MENA thoracic registrations were listed under more medically urgent statuses (eg, Pediatric Heart Statuses 1A or 2; Adult Heart Statuses 1, 2, or 3; Lung WLAUC 0–225 d, 226–288 d, 289–319 d; **Table S2, SDC**, https://links.lww.com/TXD/A879). Larger proportions of MENA registrations were listed in OPTN Region 5 (28.6% versus 11.7%), which includes Arizona, California, Nevada, New Mexico, and Utah, and OPTN Region 9 (14.5% versus 6.1%), which includes New York and western Vermont.^8^

**TABLE 1.** Demographic characteristics of MENA versus Non-MENA registrations and recipients in OPTN data.

	Registrations ever-waiting from January 1, 2018, to June 30, 2023		Transplanted recipients from January 1, 2018, to June 30, 2023	
	MENA	Non-MENA	MENA	Non-MENA
N	3114	204 022	1498	105 236
Organ (%)				
Kidney	2183 (70.1)	121 733 (59.7)	888 (59.3)	52 736 (50.1)
Liver	569 (18.3)	53 793 (26.4)	341 (22.8)	31 047 (29.5)
Thoracic	362 (11.6)	28 496 (14.0)	269 (18.0)	21 453 (20.4)
Age (%)				
<18	218 (7.0)	6815 (3.3)	139 (9.3)	4300 (4.1)
18–34	279 (9.0)	16 080 (7.9)	138 (9.2)	9082 (8.6)
35–49	560 (18.0)	38 405 (18.8)	243 (16.2)	19 471 (18.5)
50–64	1363 (43.8)	89 585 (43.9)	619 (41.3)	43 651 (41.5)
65+	694 (22.3)	53 137 (26.0)	359 (24.0)	28 731 (27.3)
Not reported	0 (0.0)	0 (0.0)	0 (0.0)	1 (0.0)
Birth sex = male (%)	2223 (71.4)	130 175 (63.8)	1051 (70.2)	67 151 (63.8)
OPTN region (%)				
1	102 (3.3)	12 575 (6.2)	43 (2.9)	5269 (5.0)
2	392 (12.6)	27 054 (13.3)	188 (12.6)	12 942 (12.3)
3	223 (7.2)	24 415 (12.0)	106 (7.1)	12 925 (12.3)
4	221 (7.1)	17 501 (8.6)	102 (6.8)	8182 (7.8)
5	890 (28.6)	23 891 (11.7)	409 (27.3)	11 988 (11.4)
6	49 (1.6)	7122 (3.5)	29 (1.9)	3890 (3.7)
7	196 (6.3)	19 263 (9.4)	98 (6.5)	10 136 (9.6)
8	66 (2.1)	15 517 (7.6)	32 (2.1)	8711 (8.3)
9	450 (14.5)	12 401 (6.1)	224 (15.0)	6444 (6.1)
10	382 (12.3)	21 528 (10.6)	188 (12.6)	11 200 (10.6)
11	143 (4.6)	22 755 (11.2)	79 (5.3)	13 549 (12.9)
Insurance status (%)				
Missing/not reported/pending	6 (0.2)	378 (0.2)	0 (0.0)	9 (0.0)
Private or self	1221 (39.2)	103 998 (51.0)	476 (31.8)	48 419 (46.0)
Public or charity	1887 (60.6)	99 646 (48.8)	1022 (68.2)	56 808 (54.0)
BMI (%)				
<18.5	176 (5.7)	7100 (3.5)	110 (7.3)	4282 (4.1)
18.5–<25	840 (27.0)	49 958 (24.5)	439 (29.3)	27 718 (26.3)
25–<30	1118 (35.9)	65 720 (32.2)	531 (35.4)	34 727 (33.0)
30–<40	914 (29.4)	74 618 (36.6)	398 (26.6)	35 785 (34.0)
40+	55 (1.8)	6172 (3.0)	19 (1.3)	2700 (2.6)
Not reported	11 (0.4)	454 (0.2)	1 (0.1)	24 (0.0)
Prior transplants = Yes (%)	257 (8.3)	18 287 (9.0)	85 (5.7)	7945 (7.5)

Waiting list metrics are at the time of listing; transplant metrics are at the time of transplant.

The Public or charity category includes Medicaid, Medicare FFS (Fee for Service), Medicare & Choice, CHIP (Children’s Health Insurance Program), Department of VA, Other government, Donation, Free Care, Foreign Government Specify, Public insurane—Medicare Unspecified, US/State Govt Agency.

Prior transplants indicate prior transplants of the same organ type.

Because of rounding, totals may not add up to exactly 100%.

BMI, body mass index, kg/m2; MENA, Middle Eastern/North African.

There were 106 734 NHW transplant recipients, of whom 1498 (1.4%) were MENA. Recipient demographics were similar to waitlist registration demographics, though there were slightly more recipients on public/charity insurance (68.2%) and differences by OPTN region (Table 1). Higher proportions of MENA recipients were transplanted at the extremes of the medical urgency distribution (**Table S3, SDC**, https://links.lww.com/TXD/A879). For example, larger proportions of MENA liver recipients were transplanted in status 1A and 1B (the most medically urgent statuses) and MELD/PELD 15–19 and MELD/PELD <15 (the least medically urgent statuses) compared with non-MENA liver recipients (**Table S3, SDC**, https://links.lww.com/TXD/A879). Similarly, larger proportions of MENA heart recipients were transplanted in Pediatric Heart Status 1A (most medically urgent), adult statuses 2 and 3 (intermediate medical urgency), and adult status 6 (least medically urgent) compared with non-MENA heart recipients (**Table S3, SDC**, https://links.lww.com/TXD/A879). Finally, higher proportions of MENA lung recipients were transplanted in the WLAUC 0–225 d (most medically urgent), WLAUC 226–288 and 289–319 d (intermediate medical urgency) categories compared with non-MENA lung recipients (**Table S3, SDC**, https://links.lww.com/TXD/A879).

Over 50% of MENA and non-MENA ever-waiting liver and thoracic registrations received a deceased donor transplant during the study period, whereas <50% of kidney ever-waiting registrations received a deceased donor transplant during the study period (**Table S4, SDC**, https://links.lww.com/TXD/A879). Median time to transplant was longer for MENA liver registrations compared with non-MENA liver registrations (662 versus 443 d; **Table S5A, SDC**, https://links.lww.com/TXD/A879). Conversely, median time to transplant was shorter for MENA thoracic registrations compared with non-MENA thoracic registrations (64 versus 83 d; **Table S5B, SDC**, https://links.lww.com/TXD/A879).

**FIGURE 1.**
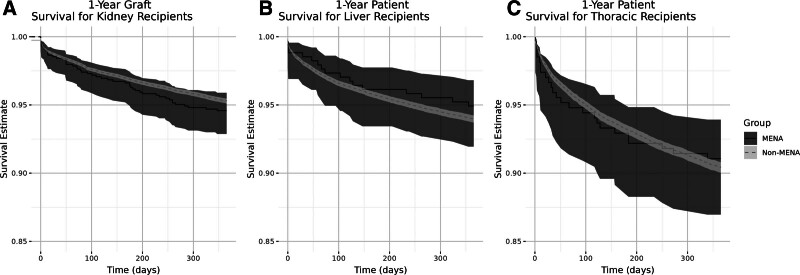
Kaplan–Meier 1-y graft or patient survival for MENA versus Non-MENA transplant recipients by organ: (A) kidney, (B) liver, and (C) thoracic.

**FIGURE 2.**
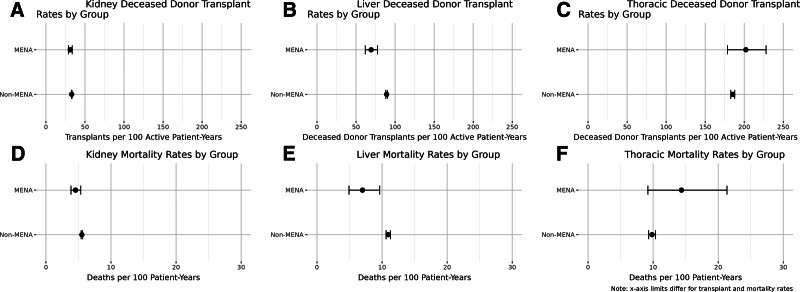
Transplant rates per 100 active patient-years (A–C) and waitlist mortality rates per 100 total patient-years (D–F) for MENA versus Non-MENA candidates by organ: (A, D) kidney, (B, E) liver, and (C, F) thoracic.

One-year graft and patient survival were similar between MENA and non-MENA groups across organs (all differences <0.01, Figure 1). Transplant and waitlist mortality rates were similar across groups for thoracic and kidney candidates but tended to be lower for MENA liver candidates as compared with non-MENA liver candidates (69.4 [CI: 61.8–77.6] versus 89.2 [CI: 88.2–90.3] deceased donor transplants per 100 active patient-years; 7.0 [CI: 4.9–9.6] versus 11.0 [CI: 10.6–11.3] waitlist deaths per 100 total patient-years; Figure 2A–F). A smaller proportion of MENA recipients received living donor transplants compared with non-MENA recipients (17.8% versus 22.1%). This held true for both kidney (16.0% versus 20.0%) and liver (1.8% versus 2.0%) recipients.

## DISCUSSION

The purpose of this study was to compare MENA and non-MENA transplant candidates and recipients before the SPD 15 revision to determine whether differences exist between these populations. We found that MENA registrations tended to be younger (with more registrations listed below age 18 y and fewer registrations listed at or above age 65 y), male, kidney registrations, with lower BMI and a higher proportion on public/charity insurance compared with non-MENA registrations. Larger proportions of MENA registrations were listed in OPTN Regions 5 and 9, consistent with the distribution of the MENA population in the 2020 US Census. MENA Census respondents tended to be younger, and California (OPTN Region 5) and New York (OPTN Region 9) had the largest proportions of MENA Census respondents.^9^

MENA kidney registrations experienced longer median dialysis time at listing than non-MENA kidney registrations, consistent with studies indicating that other minority groups are more likely to be on dialysis for longer periods of time before transplantation compared with non-Hispanic kidney candidates.^10^ Studies also indicate that while Black, Hispanic, Pacific Islander, and Indigenous Americans are more likely to be referred to transplant centers, they are less likely to be evaluated and listed^11^ because of delays in starting transplant candidacy evaluations^12^ or varying durations of these evaluations.^13^ Such observations support the creation of a distinct reporting category for MENA individuals.

Among MENA transplant recipients, a higher proportion received kidney transplants compared with liver and thoracic transplants. A similar pattern was seen among non-MENA transplant recipients, although the proportion of MENA kidney recipients was higher than that of non-MENA kidney recipients, and the proportion of MENA liver and thoracic recipients was lower than that of non-MENA liver and thoracic recipients. MENA liver candidates experienced lower transplant rates and lower waitlist mortality rates compared with non-MENA liver candidates. MENA kidney and MENA thoracic candidates, on the other hand, tended to experience similar or better transplant rates and waitlist mortality rates compared with non-MENA kidney and non-MENA thoracic candidates, respectively. MENA kidney and liver registrations were also less likely to receive living donor transplants. These differences may potentially be attributable to variability in the medical urgency and median time to transplant for MENA versus non-MENA registrations. For example, median time to transplant was longer for MENA liver registrations compared with non-MENA liver registrations (**Table S5A, SDC**, https://links.lww.com/TXD/A879), consistent with the observation that a higher proportion of MENA liver registrations were listed under the least medically urgent category. Median time to transplant for MENA thoracic registrations, on the other hand, was shorter than that for non-MENA thoracic registrations (**Table S5B, SDC**, https://links.lww.com/TXD/A879), consistent with the observation that higher proportions of MENA thoracic registrations were listed under more medically urgent statuses (**Table S2, SDC**, https://links.lww.com/TXD/A879). Finally, although median time to transplant for kidney registrations could not be calculated in this study due to the fact that <50% of kidney registrations in the ever-waiting cohort received a transplant during the study period (**Table S4, SDC**, https://links.lww.com/TXD/A879), kidney registrations are known to experience longer waiting times to transplant compared with other organs.^14^ It is important to note, however, that the sample size of MENA registrations was substantially smaller than that for non-MENA registrations, leading to more variability and greater uncertainty in estimated transplant and waiting list mortality rates, particularly for MENA thoracic candidates (Figure 2C).

These observations suggest that MENA liver candidates may be less medically urgent at listing compared with non-MENA liver candidates, potentially contributing to longer waiting times, lower transplant rates, and lower waiting list mortality rates. Conversely, other studies have suggested that non-White liver candidates experience the opposite. For example, literature indicates that Hispanic liver candidates experience an increased likelihood of waitlist removal because of death or being too sick to transplant,^15^ and that Black and Hispanic liver candidates and those on public insurance tended to be more medically urgent at listing.^16^ A potential reason for the discrepancies between our findings and those in existing literature could be due to the fact that our analyses were primarily descriptive in nature, whereas other studies employed adjusted analyses.^17-21^ As more data accrue, future research should explore whether the observed differences in medical urgency, transplantation rates, and waiting list mortality rates between MENA and non-MENA candidates persist after stratifying or adjusting for other potential confounders.

Interestingly, MENA and non-MENA recipients had comparable 1-y posttransplant graft/patient survival. Studies of Hispanic versus non-Hispanic populations yielded similar results for short-term posttransplant outcomes, though findings varied by recipients’ country of origin, geographic location, and transplant center behavior.^10^ That said, MENA recipients tended to be younger and have lower BMI at transplant. Additionally, higher proportions of MENA recipients were transplanted at the extremes of the medical urgency distribution (**Table S3, SDC**, https://links.lww.com/TXD/A879). These results suggest that the comparable short-term posttransplant graft and patient survival observed here may be partially attributable to transplanting more MENA candidates who are in overall better health compared with non-MENA candidates.

While situating our findings in the context of existing literature is important, it is crucial to note that many studies examine broader categories of race and rarely distinguish between MENA and non-MENA individuals. Such data aggregation precludes understanding of variation in health outcomes that MENA individuals may experience throughout the transplantation process.^22,23^ Our findings suggest that MENA individuals may differ from non-MENA individuals in several ways, including dialysis time at listing, medical urgency distributions, waitlist mortality rates, transplantation rates, and median time to transplant. Further research elucidating pre versus postlisting outcomes experienced by MENA individuals is necessary, especially given that factors associated with variability in referral may differ from those associated with variability in listing or transplant.^11^ Creating a new MENA category per SPD 15 guidelines represents a first step in this direction.

This study has several strengths. It is one of the first to examine demographic and clinical characteristics of MENA versus non-MENA transplant candidates and recipients in the United States. We identified key differences in clinical characteristics and outcomes that can be monitored moving forward. We also demonstrated the utility of identifying MENA individuals in OPTN data before SPD 15 revision, thereby establishing a baseline to which future analyses can be compared.

This study also has limitations. First, categorizing NHW candidates and recipients who reported Arab/Middle Eastern and North African race/ethnicity on the TCR or TRR as MENA may misclassify some non-MENA individuals as MENA or vice versa. However, this definition is consistent with SPD 15 revisions^1^ and yields comparable proportions of MENA individuals as in the 2020 US Census.^9^ Second, while OPTN TCR and TRR forms explicitly state that race/ethnicity data should be self-identified by the patient, there is no guarantee that patients themselves are providing this information. Third, MENA candidates/recipients may be more likely to select the new, separate MENA category once it becomes available,^23^ leading to changes in demographic or clinical characteristics of MENA candidates/recipients moving forward. Finally, the small sample size of MENA candidates/recipients in this study precluded adjusted analyses and analyses of longer-term posttransplant survival.

Despite these limitations, differences between MENA and non-MENA cohorts were found, suggesting that MENA individuals should be analyzed separately from NHW individuals to promote transparency.^2,3,17,22^ As SPD 15 revisions are implemented into OPTN data collection, we anticipate additional insights into the waitlist and transplantation experience of MENA candidates and recipients will be elucidated.

## Supplementary Material


