# Supplementary material for: Characterizing Middle Eastern and North African Patients in US Transplant Data
Source: Transplant Direct. 2026 Jul 7;12(8):e1973. doi: 10.1097/TXD.0000000000001973 (PMC13344926; doi:10.1097/TXD.0000000000001973)
Supplement: Supplementary file 1 [file txd-12-e1973-s001.pdf]

**Supplemental Digital Content (SDC)**

The diagrams below illustrate how inclusion/exclusion criteria were applied to OPTN Waiting List (**Figure S1A**) and OPTN Transplant (**Figure S1B**) data to obtain the final analysis cohorts for this study.

**Figure S1. Illustration of how inclusion/exclusion criteria were applied to A) waitlist registrations and B) transplant recipients to obtain the final waitlist and transplant analysis cohorts, respectively.**

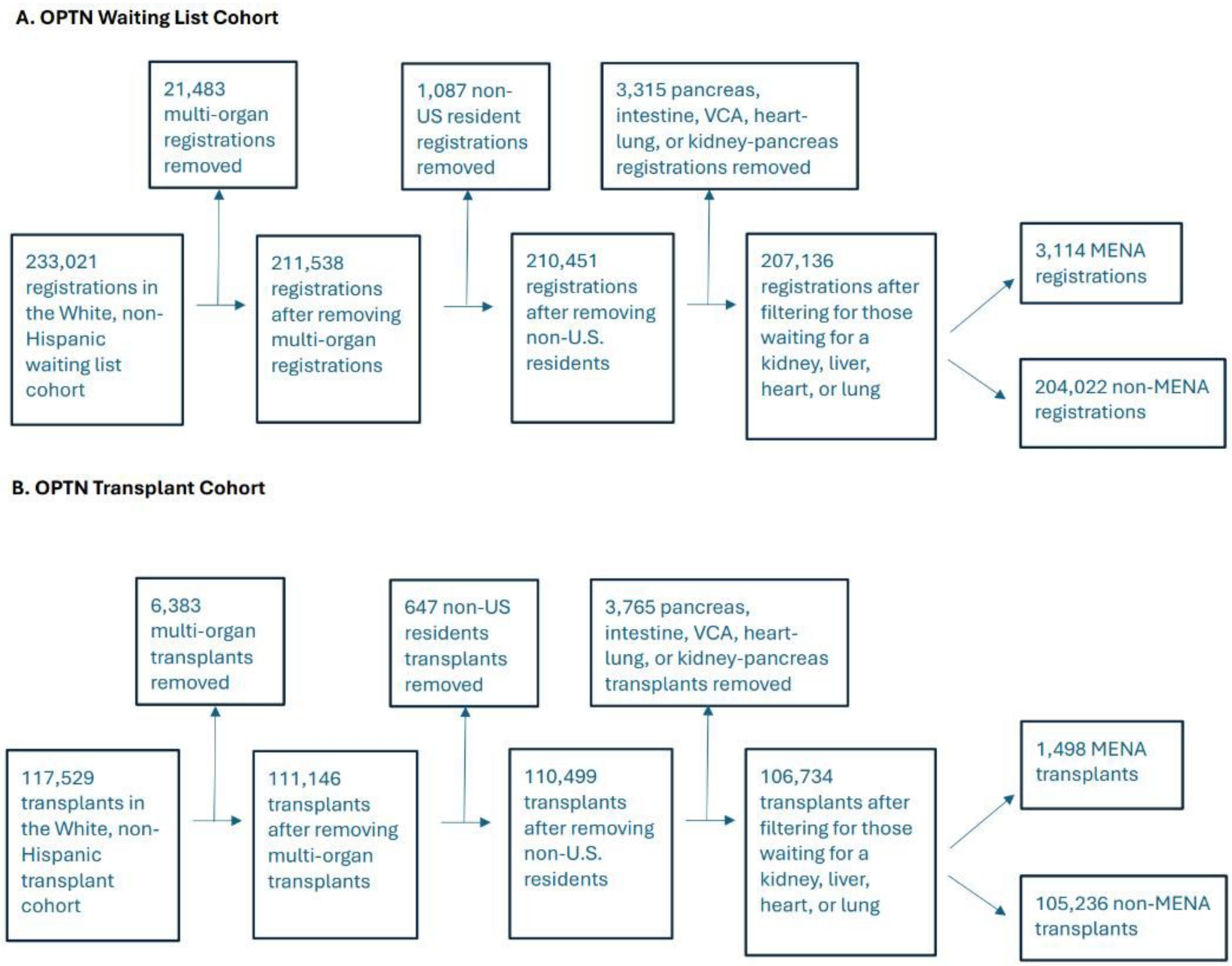


There were 233,021 registrations (117,529 transplants) in the White, non-Hispanic racial/ethnic (NHW) category, defined as those who indicated White race with no other racial group selected on the Transplant Candidate Registration Form (TCR) or Transplant Recipient Registration Form (TRR) along with either selecting "not Hispanic or Latino" or "Ethnicity not reported" for candidate ethnicity. 21,483

multi-organ registrations (6,383 multi-organ transplants) were removed as the pre- and post-transplant experience of multi-organ transplant candidates is inherently different from that of single-organ transplant candidates. Next, 1087 registrations (647 transplant recipients) who were non-U.S. residents were removed from the analyses, as the demographics of non-U.S. resident MENA candidates are inherently different from that of U.S. resident MENA candidates (**Table S1**). It is also unclear how pre- and post-transplant data collection might differ for non-U.S. residents who travel to the U.S. solely for the purposes of transplant. Thus, the non-U.S. resident MENA population is unlikely to be generalizable to the U.S. MENA population. Note that 95 registrations in the waitlist cohort and 4 recipients in the transplant cohort had missing citizenship/residency status and were also excluded from the analyses.

Subsequently, 3315 pancreas, intestine, vascularized composite allograft (VCA), heart-lung, and kidney-pancreas registrations (3765 transplants) were removed. This exclusion implies that our study is generalizable to single-organ kidney, liver, heart, or lung transplant candidates or recipients. Finally, we grouped candidates and recipients who reported Arab/Middle Eastern and/or North African race/ethnicity on the TCR or TRR within the NHW category as MENA. All other candidates and recipients in the NHW category were classified as non-MENA. The final waitlist cohort contained 3114 MENA registrations and 204,022 non-MENA registrations; similarly, the final transplant cohort contained 1498 MENA transplant recipients and 105,236 non-MENA transplant recipients. Note that the transplant cohort included all transplants that occurred between 01/01/2018-06/30/2023 and was not simply the subset of ever-waiting candidates who received transplant. Creating separate waiting list and transplant cohorts ensured that all MENA and non-MENA candidates and recipients would be captured, including those who may have indicated a different race/ethnicity on their TCR vs. TRR forms. 1243 transplants in the transplant cohort corresponded to individuals who did not meet criteria for inclusion in the waiting list cohort but who did meet criteria for inclusion in the transplant cohort. More specifically, 1195 of these individuals (96.1%) were registered for multiple organs on the waiting list but only received single-

organ transplant. The remaining 48 individuals (3.9%) were registered for kidney-pancreas on the waiting list but only received kidney transplant.

**Table S1. Demographics of MENA candidates and MENA transplant recipients by US residency.**

| | MENA Registrations Everwaiting from 01/01/2018 – 06/30/2023 | | Transplanted MENA Recipients from 01/01/2018 – 06/30/2023 | |
|---|---|---|---|---|
| | Not US resident | US resident | Not US resident | US resident |
| n | 697 | 3114 | 458 | 1498 |
| Organ (%) | | | | |
| Kidney | 232 (33.3) | 2183 (70.1) | 131 (28.6) | 888 (59.3) |
| Liver | 220 (31.6) | 569 (18.3) | 130 (28.4) | 341 (22.8) |
| Thoracic | 245 (35.2) | 362 (11.6) | 197 (43.0) | 269 (18.0) |
| Age (%) | | | | |
| <18 | 186 (26.7) | 218 ( 7.0) | 147 (32.1) | 139 ( 9.3) |
| 18-34 | 119 (17.1) | 279 ( 9.0) | 64 (14.0) | 138 ( 9.2) |
| 35-49 | 140 (20.1) | 560 (18.0) | 87 (19.0) | 243 (16.2) |
| 50-64 | 182 (26.1) | 1363 (43.8) | 108 (23.6) | 619 (41.3) |
| 65+ | 70 (10.0) | 694 (22.3) | 52 (11.4) | 359 (24.0) |
| Birth Sex = Male (%) | 429 (61.5) | 2223 (71.4) | 275 (60.0) | 1051 (70.2) |
| OPTN Region (%) | | | | |
| 1 | 17 ( 2.4) | 102 ( 3.3) | 9 ( 2.0) | 43 ( 2.9) |
| 2 | 85 (12.2) | 392 (12.6) | 50 (10.9) | 188 (12.6) |
| 3 | 38 ( 5.5) | 223 ( 7.2) | 18 ( 3.9) | 106 ( 7.1) |
| 4 | 73 (10.5) | 221 ( 7.1) | 45 ( 9.8) | 102 ( 6.8) |
| 5 | 137 (19.7) | 890 (28.6) | 83 (18.1) | 409 (27.3) |
| 6 | 1 ( 0.1) | 49 ( 1.6) | 1 ( 0.2) | 29 ( 1.9) |
| 7 | 131 (18.8) | 196 ( 6.3) | 92 (20.1) | 98 ( 6.5) |
| 8 | 4 ( 0.6) | 66 ( 2.1) | 3 ( 0.7) | 32 ( 2.1) |
| 9 | 114 (16.4) | 450 (14.5) | 88 (19.2) | 224 (15.0) |
| 10 | 79 (11.3) | 382 (12.3) | 50 (10.9) | 188 (12.6) |
| 11 | 18 ( 2.6) | 143 ( 4.6) | 19 ( 4.1) | 79 ( 5.3) |
| Insurance Status (%) | | | | |
| Missing/Not Reported/Pending | 1 ( 0.1) | 6 ( 0.2) | 0 ( 0.0) | 0 ( 0.0) |
| Private or Self | 97 (13.9) | 1221 (39.2) | 78 (17.0) | 476 (31.8) |
| Public or Charity | 599 (85.9) | 1887 (60.6) | 380 (83.0) | 1022 (68.2) |
| BMI (%) | | | | |
| <18.5 | 171 (24.5) | 176 ( 5.7) | 129 (28.2) | 110 ( 7.3) |
| 18.5-<25 | 222 (31.9) | 840 (27.0) | 143 (31.2) | 439 (29.3) |
| 25-<30 | 179 (25.7) | 1118 (35.9) | 122 (26.6) | 531 (35.4) |
| 30-<40 | 117 (16.8) | 914 (29.4) | 60 (13.1) | 398 (26.6) |
| 40+ | 6 ( 0.9) | 55 ( 1.8) | 3 ( 0.7) | 19 ( 1.3) |
| Not Reported | 2 ( 0.3) | 11 ( 0.4) | 1 ( 0.2) | 1 ( 0.1) |
| Prior Transplants = Yes (%) | 70 (10.0) | 257 ( 8.3) | 16 ( 3.5) | 85 ( 5.7) |

*Note:*
Waiting list metrics are at time of listing, transplant metrics are at time of transplant
The Public or Charity category includes Medicaid, Medicare FFS (Fee for Service), Medicare & Choice, CHIP (Children's Health Insurance Program), Department of VA, Other government, Donation, Free Care, Foreign Government Specify, Public insurance - Medicare Unspecified, US/State Govt Agency
Prior transplants indicates prior transplants of the same organ type
Due to rounding, totals may not add to exactly 100%

**Tables S2** and **S3** illustrate the medical urgency of MENA and non-MENA registrations at listing, as well as the medical urgency of MENA and non-MENA transplant recipients at transplant. Note that kidney allocation does not rely on medical urgency; as a result, no kidney medical urgency metrics are shown for the transplant cohort. However, dialysis time at listing is shown for the waiting list cohort to provide a proxy for kidney medical urgency at listing.

**Table S2. MENA vs. Non-MENA Ever-waiting Registrations from 01/01/2018 - 06/30/2023 by Medical Urgency at Listing**

| Organ | Urgency | MENA | Non-MENA |
|---|---|---|---|
| Kidney | Dialysis at Listing (Days (IQR)) | 154 (0,568) | 47 (0,437) |
| Liver | Status 1A | 18 (3.17%) | 1,018 (1.90%) |
| | Status 1B | 3 (0.53%) | 107 (0.20%) |
| | MELD/PELD 35+ | 23 (4.05%) | 4,274 (7.96%) |
| | MELD/PELD 30-34 | 21 (3.70%) | 3,736 (6.96%) |
| | MELD/PELD 25-29 | 35 (6.16%) | 4,772 (8.89%) |
| | MELD/PELD 20-24 | 67 (11.80%) | 7,090 (13.21%) |
| | MELD/PELD 15-19 | 116 (20.42%) | 11,227 (20.91%) |
| | MELD/PELD <15 | 274 (48.24%) | 20,562 (38.30%) |
| | Inactive | 11 (1.94%) | 904 (1.68%) |
| Total | - | 568 (100.00%) | 53,690 (100.00%) |
| Heart | Pediatric Heart Status 1A | 34 (13.99%) | 1,695 (10.77%) |
| | Pediatric Heart Status 1B | 24 (9.88%) | 2,133 (13.55%) |
| | Pediatric Heart Status 2 | 29 (11.93%) | 1,836 (11.66%) |
| | Adult Status 1 | 11 (4.53%) | 554 (3.52%) |
| | Adult Status 2 | 45 (18.52%) | 2,334 (14.82%) |
| | Adult Status 3 | 19 (7.82%) | 963 (6.12%) |
| | Adult Status 4 | 45 (18.52%) | 3,700 (23.50%) |
| | Adult Status 6 | 33 (13.58%) | 2,296 (14.58%) |
| | Temporarily Inactive | 3 (1.23%) | 233 (1.48%) |
| Total | - | 243 (100.00%) | 15,744 (100.00%) |
| Lung | WLAUC 0-225 days | 33 (27.50%) | 1,892 (14.36%) |
| | WLAUC 226-288 days | 26 (21.67%) | 1,690 (12.83%) |
| | WLAUC 289-319 days | 25 (20.83%) | 2,298 (17.44%) |
| | WLAUC 320-365 days | 36 (30.00%) | 7,192 (54.58%) |
| | Missing/Not Reported | 0 (0.00%) | 105 (0.80%) |
| Total | - | 120 (100.00%) | 13,177 (100.00%) |

*Note:*
Registrations having old heart and liver statuses were excluded from the waiting list portion of the table (2 heart registrations, 104 liver registrations)
Registrations with missing dialysis at listing were set to 0
Due to rounding, totals may not add to exactly 100%

**Table S3. MENA vs. Non-MENA Transplanted Recipients from 01/01/2018 - 06/30/2023 by Medical Urgency at Transplant**

| Organ | Urgency | MENA | Non-MENA |
|---|---|---|---|
| Liver | Status 1A | 16 (4.69%) | 725 (2.34%) |
| | Status 1B | 11 (3.23%) | 266 (0.86%) |
| | MELD/PELD 35+ | 63 (18.48%) | 5,878 (18.93%) |
| | MELD/PELD 30-34 | 57 (16.72%) | 5,470 (17.62%) |
| | MELD/PELD 25-29 | 57 (16.72%) | 6,483 (20.88%) |
| | MELD/PELD 20-24 | 48 (14.08%) | 4,998 (16.10%) |
| | MELD/PELD 15-19 | 51 (14.96%) | 4,291 (13.82%) |
| | MELD/PELD <15 | 38 (11.14%) | 2,928 (9.43%) |
| | Inactive | 0 (0.00%) | 8 (0.03%) |
| Total | - | 341 (100.00%) | 31,047 (100.00%) |
| Heart | Pediatric Heart Status 1A | 36 (20.69%) | 1,923 (17.49%) |
| | Pediatric Heart Status 1B | 4 (2.30%) | 682 (6.20%) |
| | Pediatric Heart Status 2 | 0 (0.00%) | 95 (0.86%) |
| | Adult Status 1 | 12 (6.90%) | 821 (7.47%) |
| | Adult Status 2 | 66 (37.93%) | 3,946 (35.90%) |
| | Adult Status 3 | 25 (14.37%) | 1,360 (12.37%) |
| | Adult Status 4 | 20 (11.49%) | 1,666 (15.16%) |
| | Adult Status 5 | 0 (0.00%) | 3 (0.03%) |
| | Adult Status 6 | 11 (6.32%) | 497 (4.52%) |
| Total | - | 174 (100.00%) | 10,993 (100.00%) |
| Lung | WLAUC 0-225 days | 39 (41.05%) | 2,558 (24.46%) |
| | WLAUC 226-288 days | 20 (21.05%) | 1,567 (14.98%) |
| | WLAUC 289-319 days | 20 (21.05%) | 1,828 (17.48%) |
| | WLAUC 320-365 days | 16 (16.84%) | 4,507 (43.09%) |
| Total | - | 95 (100.00%) | 10,460 (100.00%) |

*Note:*
Due to rounding, totals may not add to exactly 100%

**Table S4** illustrates the proportion of ever-waiting registrations between 01/01/2018 - 06/30/2023 by the proportion who received deceased donor transplant as of December 5, 2025. This table suggests that the proportion of MENA kidney registrations who received transplant was slightly higher than that of non-MENA registrations, but still noticeably below 50%. Conversely, the proportions of transplants for liver and thoracic organs were more similar between MENA and non-MENA groups and were higher than 50%.

**Table S4. MENA vs. Non-MENA Ever-waiting Registrations from 01/01/2018 - 06/30/2023 by the Proportion Receiving Deceased Donor Transplant**

| Group | Kidney | Liver | Thoracic |
|---|---|---|---|
| MENA | 769 (35.2%) | 329 (57.8%) | 281 (77.6%) |
| Non-MENA | 38,159 (31.3%) | 30,655 (57.0%) | 22,520 (79.0%) |

**Table S5** illustrates median waiting time to transplant for liver and thoracic organs (median waiting time to transplant for kidney candidates could not be calculated, as less than 50% of ever-waiting kidney registrations in our study received transplant as of December 5, 2025; see **Table S4**). Median waiting time was longer for MENA liver registrations compared to non-MENA liver registrations (**Table S5A**), consistent with the observation that a higher proportion of MENA liver registrations were listed under the least medically urgent category (i.e., MELD/PELD <15; **Table S2**). Median waiting time for MENA thoracic registrations, on the other hand, was shorter than that for non-MENA thoracic registrations (**Table S5B**), consistent with the observation that higher proportions of MENA thoracic registrations were listed under more medically urgent statuses (e.g., Pediatric Heart Statuses 1A or 2; Adult Heart Statuses 1, 2, or 3; Lung WLAUC 0-225 days, 226-288 days, 289-319 days; **Table S2**). Finally, although median waiting time for kidney registrations could not be calculated in this study due to the fact that less than 50% of kidney registrations in the ever-waiting cohort received transplant during the study period, kidney registrations are known to experience longer waiting times to transplant compared to other organs [see main text reference 14]

**Table S5. Median Waiting Time to Transplant for A) MENA vs. Non-MENA Ever-waiting Liver Registrations from 01/01/2018 - 06/30/2023, and B) MENA vs. Non-MENA Ever-waiting Thoracic Registrations from 01/01/2018 - 06/30/2023**

**A. MENA vs. Non-MENA Ever-waiting Liver Registrations from 01/01/2018 - 06/30/2023 by Median Time to Transplant**

| Group | Median Waiting Time (Days) | N Registrations |
|---|---|---|
| MENA | 662 | 569 |
| Non-MENA | 443 | 53793 |

**B. MENA vs. Non-MENA Ever-waiting Thoracic Registrations from 01/01/2018 - 06/30/2023 by Median Time to Transplant**

| Group | Median Waiting Time (Days) | N Registrations |
|---|---|---|
| MENA | 64 | 362 |
| Non-MENA | 83 | 28496 |
